# Transient spray combustion characteristics in a gas–liquid pintle rocket engine under acoustic excitation

**DOI:** 10.1038/s41598-024-64027-2

**Published:** 2024-06-07

**Authors:** Xuan Jin, Chao Zhu, Dejiang Chen, Zhongpei Zhang

**Affiliations:** 1 Hypervelocity Aerodynamics Institute, China Aerodynamics Research and Development Center, Mianyang, 621000 China; 2 National Key Laboratory of Aerospace Physics in Fluids, China Aerodynamics Research and Development Center, Mianyang, 621000 China

**Keywords:** LOX/GCH_4_ pintle engine, Transverse velocity disturbance, Acoustic response characteristic, Transient spray combustion process, Numerical simulation, Engineering, Aerospace engineering

## Abstract

In order to investigate the acoustic oscillation characteristics of gas–liquid pintle rocket engines and elucidate the path by which spray combustion process provides energy to the combustor pressure oscillation, a LOX/GCH_4_ pintle engine with rectangular combustor was designed. By adding transverse velocity disturbance for the first time, the acoustic response of spray combustion process was simulated, and the effect of excitation amplitude on acoustic response was researched. Numerical results show that the adopted transverse velocity disturbance can excite the first-order transverse acoustic oscillation with same excitation frequency in the engine combustor. The acoustic response maintenance mechanism under extrinsic excitation is summarized for pintle engines. Besides, the temperature distribution inside the engine combustor tends to be uniform, and the low-frequency oscillation caused by the flame transverse swing gradually disappears. The amplitude of combustor pressure oscillation is dominated by excitement amplitude and phase difference between the pressure and heat release in combustion reaction region. In addition, the time-averaged combustor pressure can be amplified mainly by transverse velocity disturbance. The research work can provide a reference for related fire tests on the acoustic response of a subscale gas–liquid pintle engine.

## Introduction

High frequency combustion instability is a very challenging subject^1^. Once the high frequency combustion instability occurs, the dramatically increased heat flow on the combustor wall causes the engine to burn out in a fraction of a second. High-amplitude pressure oscillation can even induce instantaneous explosion. Up to now, no consensus has been reached on the maintenance mechanism of high frequency combustion instability, and predicting combustion stability is still one of the most difficult steps in the development of new engines^2^. One reason is that the atomization, evaporation, mixing, and combustion processes are changed when different injector configurations are paired with different propellant combinations. A large number of full-scale fire tests are always used to overcome the high frequency combustion instability in engineering application, but the cost of trial-and-error method is rather high. It will be of great economic value and time benefit to explore the instability mechanism by means of theoretical analysis, numerical simulation and test verification in the engine pre-research stage. Relevant research progress can be referred to Re^3–7^.

The pintle injector, which originated in the 1950s, has many advantages such as simple structure, adjustable injection area and stable combustion^8^. The use of oxygen/methane bipropellant can achieve in-place refueling for spacecraft in future space exploration^9^. A series of researches have been carried out on the combustion stability of pintle engine at home and abroad. A series of researches have been carried out on the combustion stability of pintle engine at home and abroad, focusing on (a) recirculation zone, (b) energy release region, and (c) acoustic oscillation.

- Cheng Peng^10^ conducted experimental study on a two-dimensional engine configuration using the pintle injector with radial orifices, and believed that the head recirculation zone provides a heat source for fuel evaporation and contributed to the flame stability. Rajendran et al.^11^ found that raising the spray cone angle can make the central recirculation zone away from the injector and act as a baffle and mixer for bipropellant. By summarizing large amounts of experimental data, Dreless et al.^12^ pointed out that the recirculation zone becomes susceptible to the combustor pressure disturbance due to large gas–liquid velocity difference, which is critical for promoting the liquid-jet breakage.
- Min et al.^13^ carried out comparative study, and presented that the bipropellant reaction is completed in a conical zone near the combustor head. The drastic change of fluid physical properties (such as density, molecular weight and temperature, etc.) results in rapid dissipation or suppression of sound waves, which is conducive to combustion stability.
- Sakaki et al.^14^ found that increasing the collision area or momentum ratio of bipropellant is conducive to achieving rapid and stable ignition. The conclusion is consistent with the experimental results of ignition observation test of a pintle injection element using non-toxic hypergolic bipropellant, which was carried out by Kim et al.^15^ in atmospheric environment. Besides, it is presented from fire tests that when the total momentum ratio is less than 1, the high frequency unstable combustion process^16,17^, which is caused by the coupling of first-order longitudinal acoustic oscillation and combustion heat release, will occur in the stable oscillation stage. With the increase of the total momentum ratio, the observed oscillation frequency is equal to the reciprocal of the residence time of combustion gas in the combustor, and the spray structure and spontaneous radiation intensity have cyclic changes with the same frequency^17,18^; additional statistical results show that there is a positive correlation between oscillation intensity and combustion efficiency. Cheng^10^ revealed that the atomization performance is poor at the lowest gas–liquid ratio and momentum ratio. The head recirculation zone is in a critical flame-out state, and the flame undergoes first-order longitudinal acoustic oscillation under the influence of spray oscillation. Furthermore, Austin et al.^19^ suggested that a center-mounted pintle injector can minimize the coupling from tangential mode and first radial acoustic mode.

In general, scholars have not reached a consensus on the inherent combustion stability mechanism of pintle engine, and relevant theories lack effective verification. The research methods on the transient spray combustion characteristics of pintle injector are also limited to combustor pressure spectrum analysis, optical observation and mode decomposition of transient spray combustion process.

Compared with fire test and nonlinear theoretical analysis, more detailed flow field information can be acquired through numerical simulation of the spray combustion process. The previous simulation work^20,21^ shows that the LOX/GCH_4_ pintle engine has excellent combustion stability, and the combustor pressure oscillation has small frequency and oscillation without extrinsic excitation. At the presence of transverse velocity disturbance, the acoustic response of spray combustion is greatly affected by the relative size of the excitation frequency and the combustor natural frequency of first-order transverse oscillation mode. In this paper, the effect of excitation amplitude of transverse velocity disturbance on acoustic response characteristics is further studied.

## Physical model and numerical methodology

### Configuration

**Figure 1:**
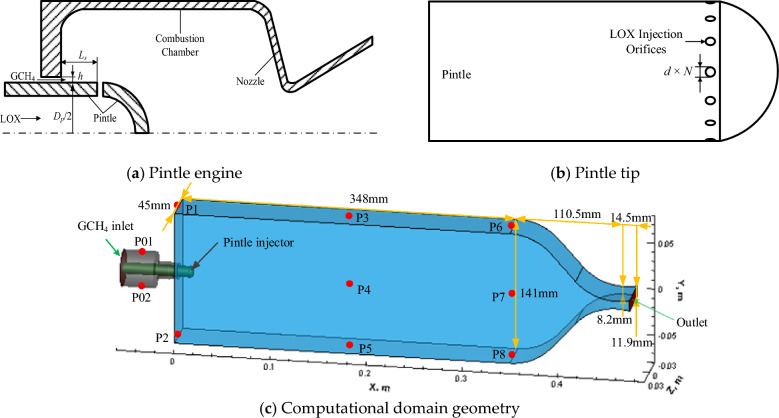
Physical model of LOX/GCH4 pintle rocket engine^21^.

The LOX/GCH_4_ pintle rocket engine prototype uses a liquid-centered gas–liquid pintle injector, and the designed combustor pressure and flow rate are 1.8 MPa and 0.356 kg/s (257 g/s for LOX, 99 g/s for GCH_4_) respectively. As displayed in Fig. 1a,b, LOX is radially injected from several injection orifices at the pintle tip after passing through the pintle central flow passage, and GCH_4_ in the gas manifold is emitted axially from the annular gap outside the pintle to form a gas film. The GCH_4_ film impinges on all radial LOX jets with an impact angle of 90° for subsequent atomization, mixing and combustion. The local momentum ratio between the radial LOX jet and the axial GCH_4_ film is set to 1. Main structural parameters of the pintle injector are as follows: L_s_ = 12 mm, D_p_ = 12 mm, h = 0.66 mm, d = 0.84 mm, N = 12.

The coupling complexity of multiple oscillation modes in a cylindrical combustor is simplified by applying unidirectional acoustic excitation in a rectangular combustor. The computational domain used for numerical simulation is shown in Fig. 1c. The origin of computational domain is the cross-section center point of the combustor inlet cross-section. The rectangular combustor has the length (x-direction) of 348 mm, the height (y-direction) of 141 mm, and the width (z-direction) of 45 mm. The nozzle height is first shrunk to 8.2 mm at the throat and then extended to 11.9 mm at the outlet. Multiple pressure monitoring points are set on the z = 0 plane during unsteady numerical simulations, where P4 and P7 are located on the combustor axis and the others on the wall. The X-axis coordinates corresponding to each point are as follows: − 28 mm(P01/P02), 0(P1/P2), 174 mm(P3/P4/P5), 174 mm(P6/P7/P8).

### Numerical method

The Euler–Lagrange method is adopted to calculate two-phase flow inside the LOX/GCH_4_ pintle rocket engine by means of a commercial code FLUENT^22^. The governing equations include unsteady Reynolds-averaged Navier–Stokes equations in Euler frame (for continuous phase) and discrete particle trajectory equations in Lagrange frame (for discrete phase). The coupling between continuous and discrete phases is realized by the gas–liquid interaction source term. The governing equations are discretized by the finite volume method, and then solved by the density-based implicit algorithm.

In the Euler frame, the density-weighted filtered governing equations for continuous phase can be written as^23^

1 $$\frac{{\partial \overline{\rho }}}{\partial t} + \frac{{\partial \user2{(}\overline{\rho }\tilde{u}_{i} \user2{)}}}{{\partial x_{i} }} = \rho_{s}$$

2 $$\frac{{\partial \user2{(}\overline{\rho }\tilde{u}_{i} \user2{)}}}{\partial t} + \frac{{\partial \user2{(}\overline{\rho }\tilde{u}_{i} \tilde{u}_{j} \user2{)}}}{{\partial x_{j} }} = \frac{{\partial \user2{(}\overline{\tau }_{ij} \user2{ - }\overline{{\rho u^{\prime\prime}_{i} u^{\prime\prime}_{j} }} \user2{)}}}{{\partial x_{j} }} - \frac{{\partial \overline{p}}}{{\partial x_{j} }} + F_{s,i}$$

3 $$\frac{{\partial \user2{(}\overline{\rho }\tilde{E}\user2{)}}}{\partial t} + \frac{{\partial \user2{(}\overline{\rho }\tilde{E} + \overline{p}\user2{)}\tilde{u}_{i} }}{{\partial x_{i} }} = \frac{{\partial \user2{(}\overline{{\tau_{ij} u_{j} }} \user2{ - }\overline{{q_{i} }} - \overline{{u^{\prime\prime}_{i} p}} - \overline{{\rho u^{\prime\prime}_{j} \user2{E^{\prime\prime}}}} \user2{)}}}{{\partial x_{i} }} + S_{h} + Q_{s}$$

4 $$\frac{{\partial \user2{(}\overline{\rho }\tilde{Y}_{m} \user2{)}}}{\partial t} + \frac{{\partial \user2{(}\overline{\rho }\tilde{u}_{j} \tilde{Y}_{m} \user2{)}}}{{\partial x_{i} }} = \frac{\partial }{{\partial x_{i} }}\user2{(}\frac{{\mu_{t} }}{{Sc_{t} }}\frac{{\partial \tilde{Y}_{m} }}{{\partial x_{i} }}\user2{) + }\frac{\partial }{{\partial x_{i} }}\user2{(}\overline{\rho }\tilde{D}_{m} \frac{{\partial \tilde{Y}_{m} }}{{\partial x_{i} }}\user2{)} - \omega_{m} + S_{s,m}$$

where the over bars (–) and quotes (") respectively represent the time filtered values and their pulsation values, the over tildes (~) and quotes (') respectively represent the density-weighted filtered values and their pulsation values, t is the physical time, *ρ* is the density, and u_i_ and q_i_ are the velocity and heat flux vector in i-direction (i = 1, 2 and 3 represent x, y, and z-direction in Cartesian coordinate system, respectively), τ_ij_ is the viscous stress, *p* is the pressure, E is the total energy, and D_m_ and Y_m_ are the diffusion coefficient and mass fraction of species m, μ_t_ is the turbulent viscosity coefficient (solved by introducing the standard k − ε turbulence model), Sc_t_ is the Schmitt number, S_h_ and ω_m_ are the source terms due to chemical reaction, ρ_s_, F_s,i_, Q_s_ and S_s_, are the interphase exchange terms provided by the discrete phase.

In the Lagrange frame, the primary and secondary breakup processes of liquid jets are not taken into consideration. For simplicity, the atomization process of liquid jets is modeled as droplet particles by means of the Discrete Phase Model (DPM). LOX particles are injected into the computational domain from injection surfaces, and no interaction between particles is yet considered. The governing equations for discrete phase can be expressed as particle trajectory equations^24^, and unsteady Lagrange particle tracking method is used to describe the motion and evaporation of each particle. The Spherical Drag Law is adopted without considering the non-sphericity of droplet shape. The interaction of the droplet with combustor walls utilized the reflect type boundary condition with all normal and tangential momentum retained. During the calculation process, required information of the surrounding gas is provided by the governing equations for continuous phase, while the gas source terms are calculated and delivered to the latter.

The droplet motion equation is

5 $$\frac{{d^{2} {\mathbf{x}}}}{{dt^{2} }} = \frac{{d{\mathbf{u}}_{{\mathbf{p}}} }}{dt} = F_{D} \left( {{\mathbf{u}}_{{\mathbf{g}}} - {\mathbf{u}}_{{\mathbf{p}}} } \right) + {\mathbf{g}}\left( {1 - \rho_{g} /\rho_{p} } \right)$$

The droplet evaporation rate is

6 $$- \frac{{dm_{p} }}{dt} = k_{c} A_{p} \rho_{g} B_{M}$$

The droplet temperature change is determined by the latent heat of evaporation and surface convective heat transfer:

7 $$m_{p} \cdot c_{p} \cdot \frac{{dT_{p} }}{dt} = hA_{p} \left( {T_{g} - T_{p} } \right) + \frac{{dm_{p} }}{dt}h_{fg}$$

The governing equation of droplet diameter is

8 $$\frac{d}{dt}d_{p} = - \frac{{d_{p} }}{3\Delta t}\frac{{\Delta \rho_{p} }}{{\rho_{p} }} - \frac{{2\dot{m}}}{{\pi d_{p}^{2} \rho_{p} }}$$

where **u** is the velocity vector, **g** is the acceleration vector, F_D_ is the drag force coefficient, m_p_ is the droplet mass, k_c_ is the mass transfer coefficient, A_p_ is the droplet surface area, B_M_ is the Spalding mass transfer number, c_p_ is the specific heat, h is the convective heat transfer coefficient, T is the temperature, h_fg_ is the latent heat of evaporation, d_p_ is the droplet diameter, the subscripts p and g respectively represent the droplet and its surrounding gas.

Mass, momentum and energy exchange occurs at the interface between continuous and discrete phases. Suppose that the droplet number contained in a droplet cluster is n_p_, and the number of droplet clusters passing through a grid cell with the volume of ΔV is ψ. When these droplet clusters pass through this grid cell, the interphase exchange terms generated by the discrete continuous phase in Eqs. (1)–(4) can be expressed as

9 $$\left\{ \begin{gathered} \rho_{s} \hfill \\ F_{s,i} \hfill \\ Q_{s} \hfill \\ S_{s,m} \hfill \\ \end{gathered} \right\} = \frac{1}{\Delta V}\left\{ \begin{gathered} \sum {_{\psi } \user2{(}n_{p} \dot{m}_{p} \user2{)}} \hfill \\ \sum {_{\psi } n_{p} \left[ {\dot{m}_{p} u_{i,p} \user2{ - }\frac{{\pi \rho_{p} d_{p}^{3} }}{6}\frac{{du_{i,p} }}{dt}} \right]} \hfill \\ \sum {_{\psi } n_{p} \left[ {\dot{m}_{p} h_{gf} \user2{ + }h_{p} \pi d_{p}^{2} \user2{(}T_{p} \user2{ - }T_{g} \user2{) - }m_{p} u_{i,p} \frac{{du_{i,p} }}{dt} + \frac{{\dot{m}_{p} u_{i,p}^{2} }}{2}} \right]} \hfill \\ \sum {_{\psi } \user2{(}n_{p} \dot{m}_{p} \user2{)}} \hfill \\ \end{gathered} \right\}$$

The actual combustion process is influenced by chemical reaction kinetics, molecular transport and turbulent mixing.

In order to solve the chemical-reaction source terms S_h_ and ω_m_ in Eqs. (3)–(4), the eddy dissipation concept model (EDC) is applied for turbulent combustion simulation, and the reaction rate of every reaction is calculated individually by formulating small-scale eddies. Giorgi et al.^25^ compared multiple turbulent combustion models for the coaxial rocket engine, and revealed that the EDC was numerical cheaper to predict the combustor temperature and flame length.

10 $$S_{h} = - \sum\limits_{m = 1}^{{N_{sp} }} {\omega_{m} M_{m} } h_{m}$$

11 $$\omega_{m} = \frac{{\rho \left( {\xi^{ * } } \right)^{2} }}{{\tau^{ * } \left[ {1 - \left( {\xi^{ * } } \right)^{3} } \right]}}\left( {Y_{m}^{ * } - Y_{m} } \right)$$

where N_sp_ is the total number of species, H_m_ is the enthalpy of species m, Y_m_^*^ is the mass fraction of species m in a small-scale eddy, ξ^*^ is the dimensionless scale of a small-scale eddy, τ^*^ is the reaction time scale of species m in a small-scale eddy.

Considering the limited computational resources, the Jones-Lindstedt 4-step mechanism (JL) is used to model real chemical reactions, as listed in Table 1. Andersen et al.^26^ combined the JL with the EDC and provided an accurate temperature field and CO distribution under the oxygen-methane condition.

**Table 1:** Jones–Lindstedt global multi-step methane combustion mechanism^26^.

No	Reactions	Pre-exponential factor	Temperature exponent	Activation energy (cal/mol)	Reaction orders
JL1	CH_4_ + 0.5O_2_ ⟹ CO + 2H_2_	7.82 × 10^13^	0	30.0 × 10^3^	[CH_4_]^0.5^[O_2_]^1.25^
JL2	CH_4_ + H_2_O ⟹ CO + 3H_2_	0.30 × 10^12^	0	30.0 × 10^3^	[CH_4_][H_2_O]
JL3	H_2_ + 0.5O_2_ ⟺ H_2_O	1.21 × 10^18^	− 1	40.0 × 10^3^	[H_2_]^0.25^[O_2_]^1.5^
JL4	H_2_O + CO ⟺ H_2_ + 0.5O_2_	2.75 × 10^12^	0	20.0 × 10^3^	[H_2_O][CO]

### Simulation setup

For the computational domain in Fig. 1c, the characteristic inflow condition with constant temperature (300 K) and mass flow rate (257 g/s) is utilized at the inlet of GCH_4_. According to the discrete phase model (DPM), each LOX injection orifice surface at the pintle head (see Fig. 1b) is used as injection source with the same mass flow rate (8.25 g/s). The particle type is chosen as Droplet with given initial temperature (80 K), velocity (33.48 m/s, corresponding to the injection pressure drop of 1 MPa) and diameter (10 μm), and the particle injection direction is perpendicular to the injection orifice surface. The adiabatic, nonslip wall will reflect the LOX droplets after collision. The outlet boundary condition is extrapolated by numerical simulation. In particular, the selection of droplet size is based on previous simulation study^20^: There is a negative correlation between the initial LOX droplet diameter and combustion efficiency until the combustion terminates with the initial LOX droplet diameter greater than 110 μm. In order to avoid the influence of combustion efficiency on the acoustic response, the droplet size is selected as 10 μm.

Due to the irregular configuration of the computational domain, structural grid is used for meshing gas manifold and annular gap, and unstructured grid for the combustor and nozzle. The structural and unstructural grid are spliced on the exit surface of the annular gap. In order to better resolve the flames and shear layers, the unstructured grid part is generated by bottom-up method: Firstly, surface grid with different densities are generated at different boundaries; Secondly, the boundary layer grid is generated based on the surface grid of the pintle head; Thirdly, the rest space is filled with locally encrypted volume grid. The whole computing domain contains 740,000 grid units, which meet the requirements of standard k-ε turbulence model and wall function, and the time step is set to 5 × 10^-6^ s.

**Figure 2:**
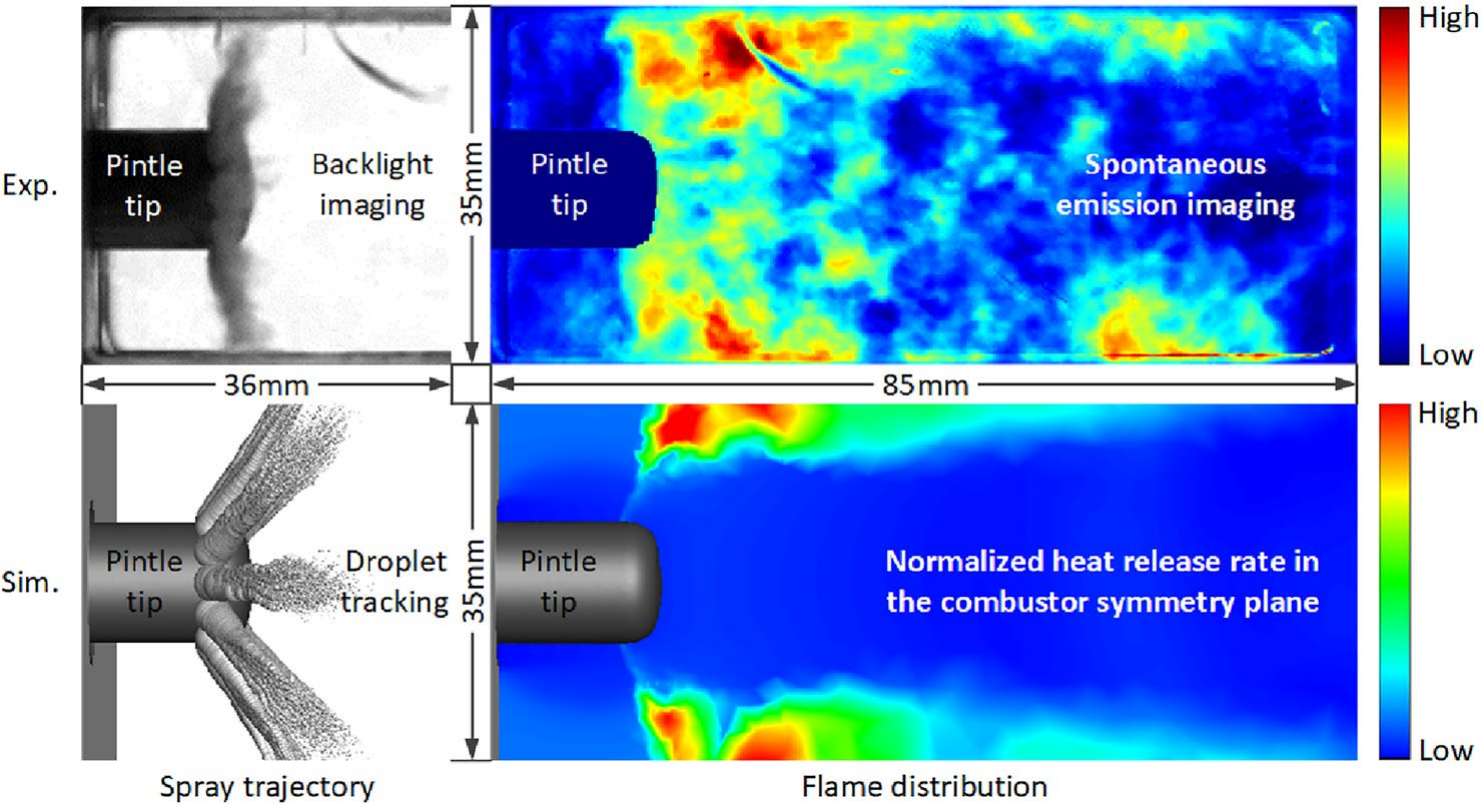
Comparison of experimental and simulation results on spray trajectory and flame distribution^21^.

The open-window fire test results of LOX/GCH_4_ pintle engine are used to verify the numerical method. Correspondingly the annular gap width (h) and injection orifice diameter (d) are respectively 2.23 mm and 0.56 mm, and the corresponding flow rates of GCH_4_ and LOX are respectively 36.2 g/s and 95.3 g/s. Other structure parameters and simulation settings are consistent with those mentioned above. A single-camera scheme for simultaneous observation of spray (background light imaging) and flame (spontaneous radiation imaging) is adopted during the open-window fire test. The instantaneous spray trajectory and flame distribution near the pintle tip in the stable combustion stage are shown in Fig. 2. The comparison shows that the LOX particle distribution from numerical simulation is similar to that from fire test, and the evaporation processed are both completed in the vicinity of the pintle tip. Besides, the most intense burning occurs in the outer spray edge within the observation window scope, for which the numerical method and computational mesh are proved to be reasonable.

For more details of the simulation verification in this subsection, the reader should consult Ref.^20^.

### Acoustic excitation method and validation

**Figure 3:**
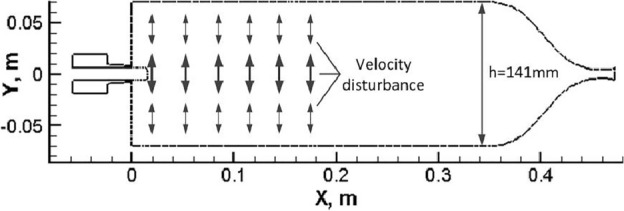
Setup of transverse velocity disturbance^21^.

Referring to the idea of rotating gears stimulating acoustic disturbance from Refs.^5,27^, the calculation order in this paper is: the stable combustion state without extrinsic excitation is firstly acquired by the aforementioned simulation setup, and then the transverse velocity disturbance is added for further researching the acoustic response of spray combustion process. The combustion flowfield without extrinsic excitation can be found in Ref.^21^: The mixing ratio O/F distribution in the combustor is very uneven, and the main reaction zone and high temperature gas mostly gather in the first half section of combustor. Thus, the presence of acoustic excitation will further promote the reaction between methane and oxygen, especially for the first half section of combustor. According to the simulation researches of Hakim^28^ and Yuan^4^, the specific setup of transverse velocity disturbance (y-direction) shown in Fig. 3 can be expressed as Eq. (12).

12 $$v^{\prime} = v^{\prime}_{0} {\varvec{sin}}\left( {\frac{{{\varvec{h}} - 2y}}{{{\varvec{2h}}}}\pi } \right)\user2{cos(}2\pi ft\user2{)}$$

Appropriate velocity disturbance in y-direction is generated by a harmonically fluctuating source term Mom' in the momentum transport equation perpendicular to the main flow direction, and always has the same phase in the entire computational domain. A momentum source term is a volumetric force and thus can be calculated as the product of density and acceleration. The acceleration is again obtained from the time derivative of the velocity fluctuation. By this approach, the following expression for the momentum source term Mom', i.e. the momentum change rate in the grid cell, is introduced into the transport conservation equation by loading a user-defined function.

13 $$Mom^{\prime} = \frac{{d\user2{(}\rho v^{\prime}\user2{)}}}{{d\user2{(}t\user2{)}}} \approx 2\pi f \cdot \rho \cdot v^{\prime}_{0} \cdot {\varvec{sin}}\left( {\frac{{2y - {\varvec{h}}}}{{{\varvec{2h}}}}\pi } \right)\user2{sin(}2\pi ft\user2{) , }x \in \left[ {0{\varvec{m}}, 0.174{\varvec{m}}} \right]$$

where v_0_′ represents the excitement amplitud, h represents the combustor height (141 mm), f represents the excitement frequency (set to 4538 Hz, which is the combustor natural frequency of first-order transverse oscillation mode), ρ represents the combustion gas density.

As the phase difference of pressure and heat release rate is strongly related to excitation amplitude, this paper places emphasis on the dynamic response of spray combustion to different excitement amplitudes at the same excitement frequency. The main focus is on three values (30 m/s, 60 m/s and 90 m/s), which reveals three typical oscillating processes of combustor pressure.

Additionally, the one-dimensional acoustic oscillation in a closed square cavity is solved theoretically and compared with the two-dimensional simulation results for acoustic validation. The length L and width w of the rectangular closed square cavity are 1 m (− 0.5 m ≤ x ≤ 0.5 m) and 0.01 m (0 ≤ y ≤ 0.01 m), respectively. The cavity is filled with still air (pressure p_0_ is 1.8 MPa, temperature is 300 K, density ρ is 21 kg/m^3^, gas constant is 286.96 J/(kg K), specific heat ratio is 1.4, sound velocity c is 347.19 m/s). The velocity field (v_x_ = 10·sin[(0.5 + x)·π]) is given at time t = 0, then the time history of pressure oscillation at x = 0.5 m is recorded. According to the first-order longitudinal oscillation mode within the square cavity (natural frequency f_c_ = c/2L = 173.6 Hz), the theoretical time history of pressure oscillation at x = 0.5 m is as follows:

14 $$p = p_{0} - 10\rho \cdot c \cdot {\varvec{cos}}\left[ {\left( {0.5 + x} \right) \cdot {\varvec{\pi}}} \right] \cdot {\varvec{sin}}\left( {2{\varvec{\pi}} \cdot f_{{\varvec{c}}} \cdot t} \right)$$

**Figure 4:**
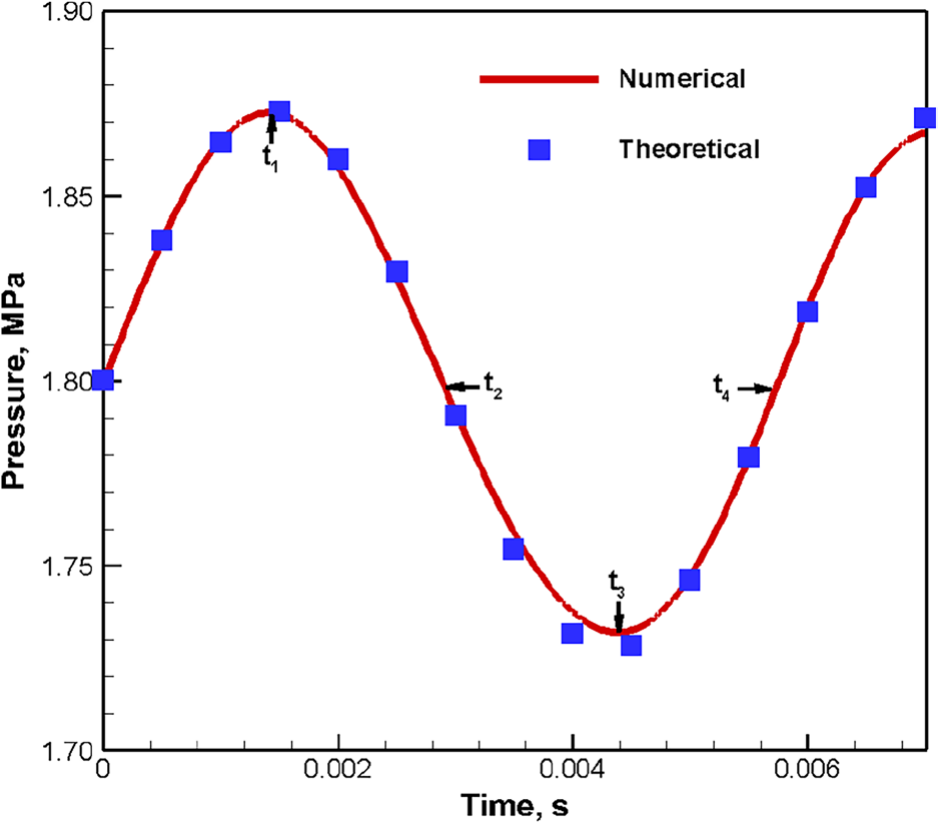
Time history of pressure oscillation at x = 0.5 m.

**Figure 5:**
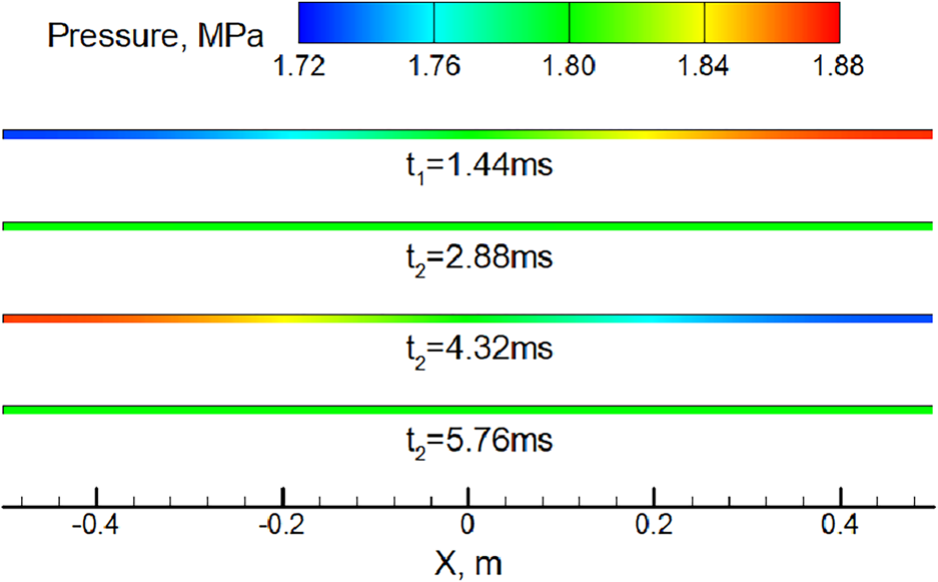
Evolution of cavity pressure field in an oscillating period. The time of each snapshot corresponds to that shown in Fig. 4.

The simulated time history of area-weighted pressure at x = 0.5 m and the theoretical solution are compared in Fig. 4, and their oscillation waveform and frequency are basically the same. The main difference is that the pressure oscillation amplitude of the former decreases significantly with the development of time, because the theoretical solution does not take into account the viscous dissipation and other factors. According to the numerical simulation result, the evolution of cavity pressure field within one oscillation period (see Fig. 5) conforms to the first-order longitudinal oscillation mode. Therefore, the calculation method proposed in this paper is suitable for numerical simulation of acoustic oscillation.

## Results and discussion

### Influence of excitement amplitude on combustor pressure oscillation

**Figure 6:**
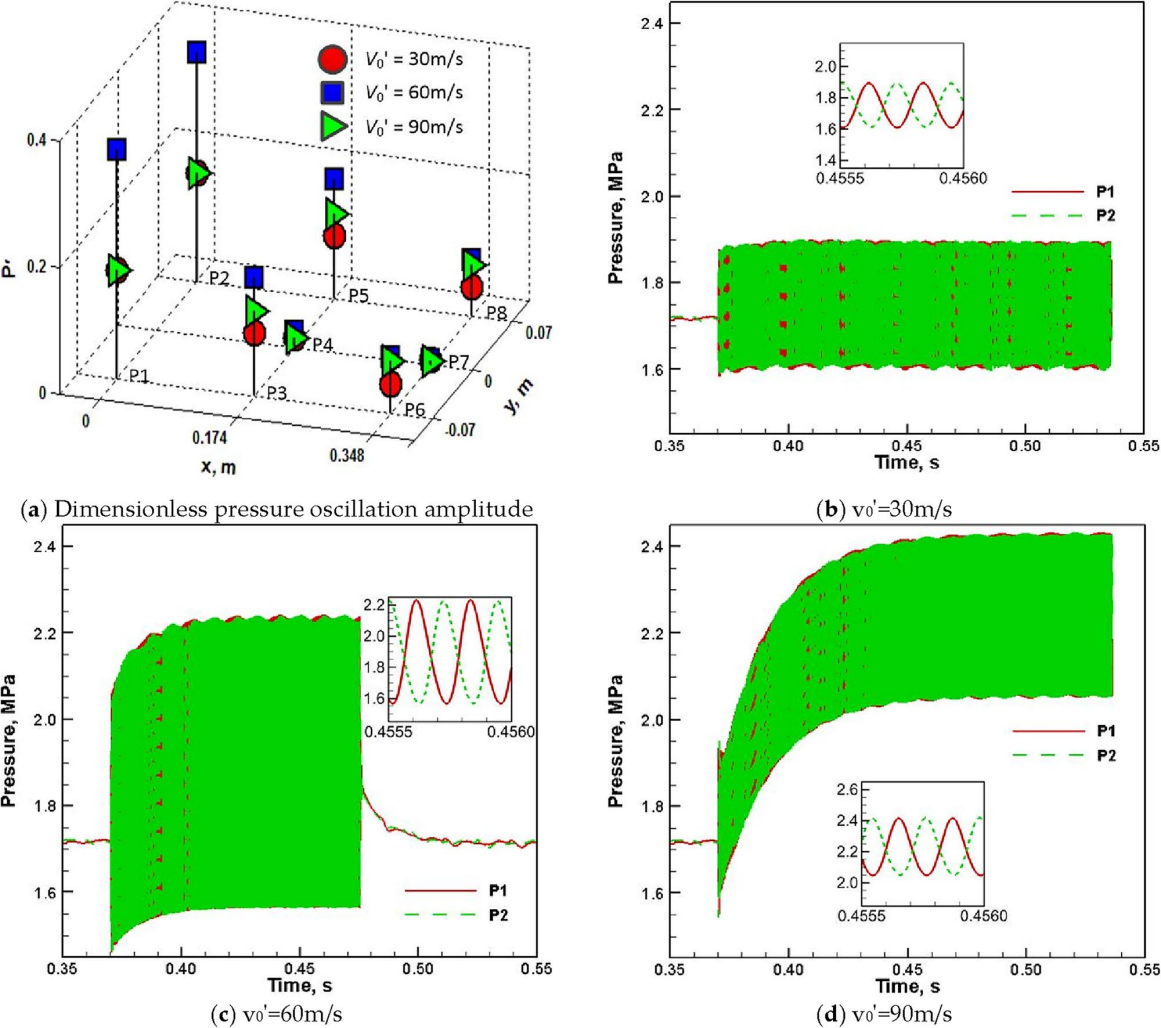
Combustor pressure oscillation under different excitement amplitudes.

When the acoustic excitation is turned on at t = 370.35 ms, the dimensionless pressure oscillation amplitude of each pressure monitoring point in the combustor under different excitement amplitudes (30 m/s, 60 m/s and 90 m/s)is displayed in Fig. 6a. There is a nonlinear relationship between the pressure oscillation amplitude and the excitement amplitude, and the combustor pressure oscillation response is most severe when v_0_′ = 60 m/s. The corresponding pressure time histories of the monitoring points (P1 and P2) located at the head of the combustion chamber are presented in Fig. 6b–d. With the increase of the excitation amplitude, the time interval from the occurrence of high-frequency combustor pressure oscillation to the stable oscillation waveform gradually increases, and the time-averaged combustor pressure of the stable oscillation waveform also rises correspondingly. Combined with the local magnification diagram of the stable oscillation waveform in Fig. 6b–d, it can be seen that the change of the excitement amplitude does not alter the first-order transverse oscillation mode and oscillation frequency of the combustor pressure. In addition, once the transverse velocity disturbance is turned off at t = 475.35 ms, the combustor pressure begins to decline and its oscillation disappears rapidly (see Fig. 6c). After about 50 ms, the pressure oscillation waveform returns to the non-excited state. The combustor pressure time history shown in Fig. 6c indicates that the pintle engine has a strong dissipation effect on the combustor acoustic oscillation, and additional measures (such as the momentum source term introduced in this paper) are essential to maintain the strong coupling between spray combustion and acoustic oscillation.

### Response of spray and combustion flowfield

**Figure 7:**
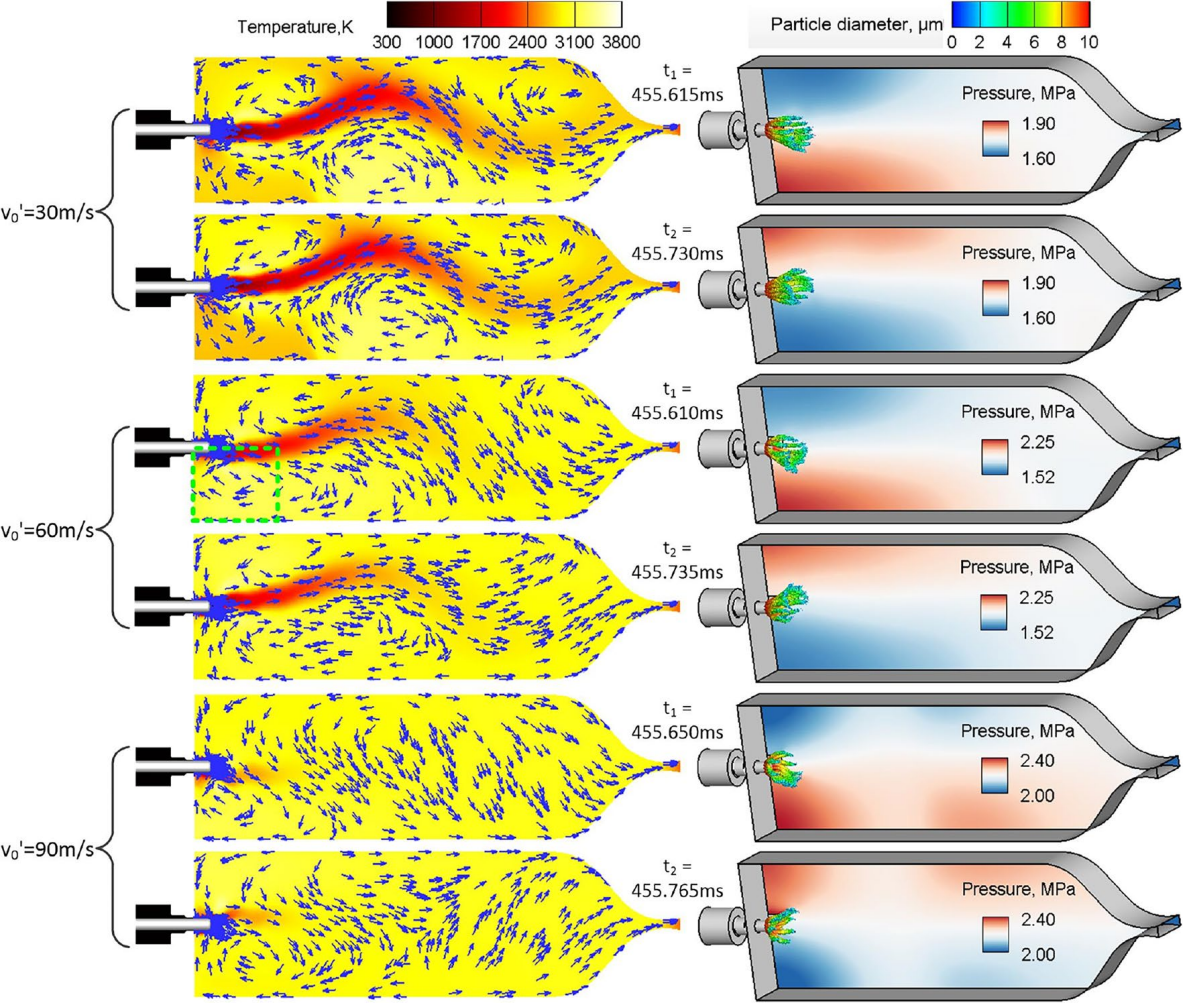
Snapshots of combustion flow field with an interval of about half a period of first-order transverse pressure oscillation for different excitement amplitudes: the left column shows the longitudinal slice of temperature, superimposed on the contour are velocity vectors represented by blue arrows; the right column shows the instantaneous pressure field in z = 0 plane and the distribution of LOX particles.

The difference of dynamic acoustic response of spray combustion under different excitation amplitudes is explained by combustion flowfield. Figure 7 exhibits the combustion flowfield of two adjacent extreme pressure points under different excitement amplitudes, which have a time interval of about half a high-frequency pressure oscillation period as seen from the local magnification in Fig. 6. It can be seen that the relative pressure changes near the upper and lower combustor walls under the acoustic excitation are consistent with the first-order transverse mode. The acoustic excitement can promote the liquid oxygen evaporation and mixing reaction between oxygen and methane in the front part of combustor (For more explanation, the reader should consult Ref.^21^). As the length of low temperature zone is gradually shortened, the combustor temperature distribution tend to be uniform.

**Figure 8:**
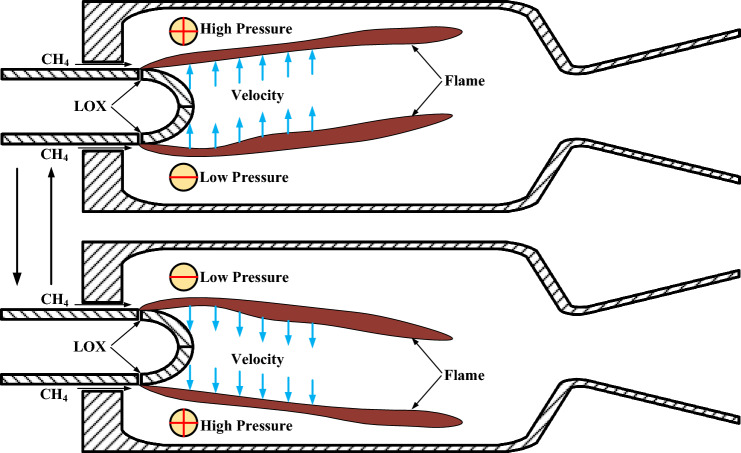
Schematic diagram of acoustic oscillation response maintenance principle under extrinsic excitation.

As shown in Fig. 8, the acoustic response maintenance mechanism under extrinsic excitation can be summarized as follows: The combustion flame oscillates periodically with acoustic excitation to maintain the high-frequency unstable combustion. When the transverse velocity disturbance blows from the lower part of the combustion chamber to the upper part, the high-temperature combustion gas and combustion flame move towards to the upper part, and the combustion heat release process will generate energy to amplify the pressure in this region. Then, a high pressure area is formed near the upper combustor wall, and a low pressure area near the lower combustor wall. Due to the direction reversal of transverse velocity disturbance and the pressure difference between the upper and lower walls, the high-temperature combustion gas and combustion flame are gradually blown from the upper part of the combustion chamber to the lower part. The pressure near the lower and upper walls respectively go through a gradual rise and fall. The repeated occurrence of the above process maintains the pressure oscillation and unsteady combustion within the pintle engine combustor.

According to the Rayleigh index (see Eq. (15)) about the interaction of combustion with transverse velocity fluctuations in a subscale liquid rocket engine^29^, the flame moving towards the high pressure region amplifies the combustor pressure oscillation, and the corresponding Rayleigh index R_f_ remains positive.

15 $$R_{f} = \frac{1}{{t_{f} - t_{0} }}\int_{{t_{0} }}^{{t_{f} }} {\overline{Q} \cdot \Delta^{\prime} \cdot \frac{{\partial p^{\prime}}}{\partial y}dt}$$

where t_0_ and t_f_ are respectively the start and end times of the integration, Δ′ is the acoustic displacement, Q is the heat release rate, əp′/əy is the gradient of pressure fluctuation field near the combustor axis.

**Figure 9:**
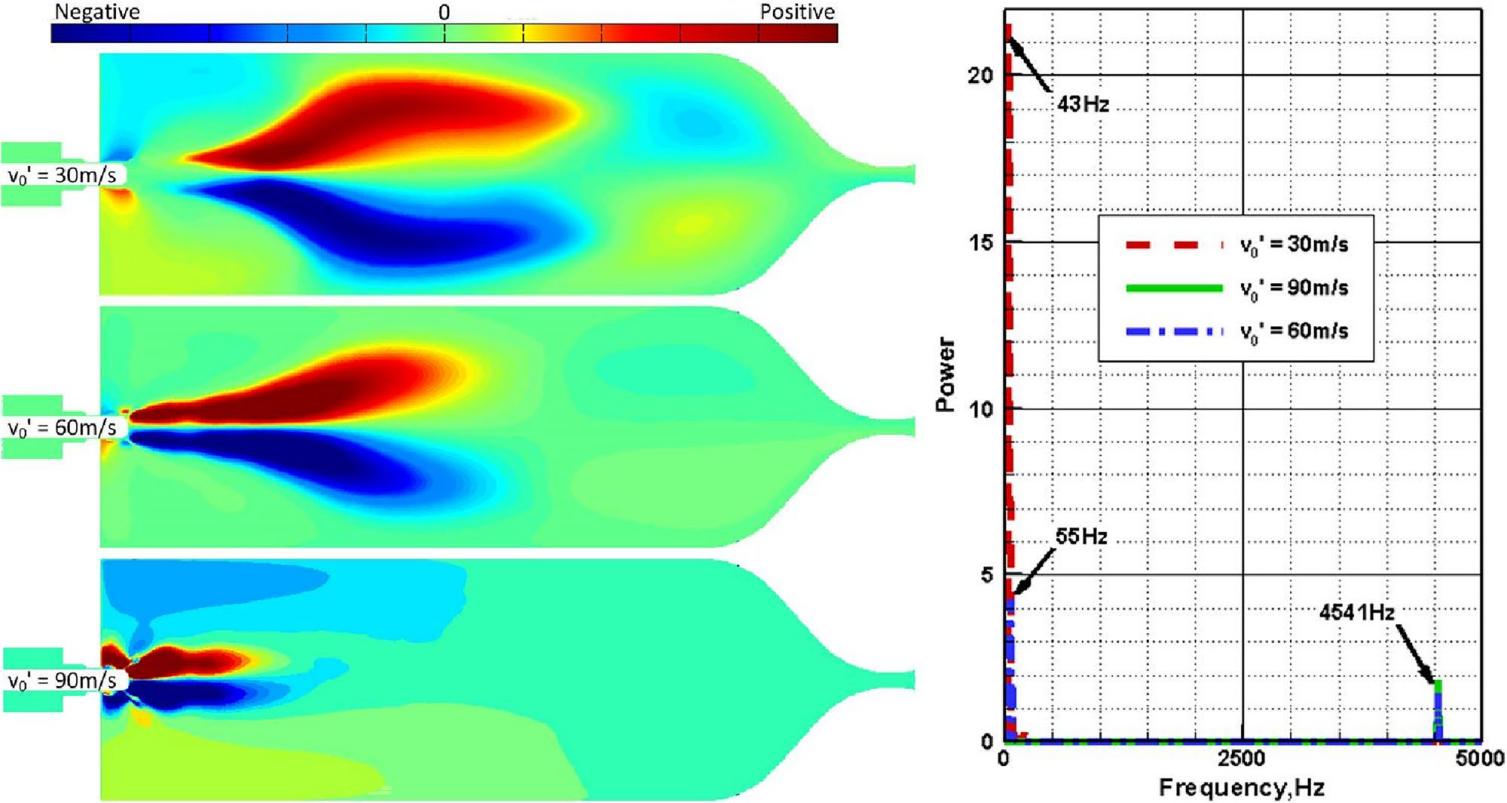
The spatial structures (left) of the dominant POD modes and their power spectrums (right) of the time coefficients under different excitement amplitudes.

In order to perform a quantitative analysis of the combustor temperature field, the proper orthogonal decomposition (POD) method summarized briefly in the Appendix, is employed to separate the time and space information for the evolution of temperature distribution on the symmetry plane of z = 0 as displayed in the left column of Fig. 7. As revealed in Fig. 9, the spatial structure of each dominant POD mode, which have the largest energy contribution on the temperature field, can be represented as the flame swing in y direction under different excitement amplitudes. With the increase of excitement amplitude, the combustion reaction and temperature field uniformity are significantly enhanced. Accordingly, the spatial structure of the dominant POD mode has a decreasing area with dramatic temperature change, and the amplitude of the main oscillating frequency in the time coefficient power spectrum also declines significantly. Moreover, the driving effect of transverse velocity disturbance with larger excitement amplitude on high temperature gas is more intense. The time coefficient of dominant POD mode changes from low-frequency oscillation (v_0_′ = 30 m/s) to mixed low/high-frequency oscillation (v_0_′ = 60 m/s). The high frequency oscillation occupies a dominant position when v_0_′ = 90 m/s, and the low-frequency oscillation basically disappears. According to Refs.^20,21^, when the excitement amplitude is small, the density and axial injection velocity of LOX and GCH_4_ inflow are much higher than those of high-temperature gas respectively. Thus, the acoustic excitation has little influence on them, so the low-frequency oscillation caused by the self-sustained flame swing in y direction always exists.

### Acoustic characteristics analysis

In the stable oscillation stage under different excitement amplitudes, the flowfield region within the cyan dashed box (0 ≤ x ≤ 0.07 m, − 0.07 m ≤ y ≤ 0) in Fig. 7 is integrated to solve the area-weighted pressure and heat release rate. The main purpose of choosing such integral region is to compare the phase relationship between pressure and heat release rate within acoustic excitation region, and then explain the differences of combustor acoustic oscillation under different excitement amplitudes using the acoustic power balance equation, (as shown in Eq. (16), see Ref.^30^ for the specific derivation and explanation).

16 $$\frac{\partial e}{{\partial t}} = \frac{{\partial \left( {\frac{1}{2}\overline{\rho } {\mathbf{u}}^{{\prime}{2}} + \frac{1}{2}\frac{{p^{{\prime}{2}} }}{{\overline{\rho } c^{2} }}} \right)}}{\partial t} = S - \nabla \cdot \left( {p^{\prime}{\mathbf{u}}^{\prime}} \right) - D$$

where e represents the acoustic power, p′u′ represents the acoustic velocity, acoustic source term S mainly contains the acoustic excitation term S_1_ and the combustion heat release term S_2_ (S_2_ = p′·Q′·(k − 1)/(k·—)), k represents the specific heat ratio, D represents the acoustic dissipation termp.

**Figure 10:**
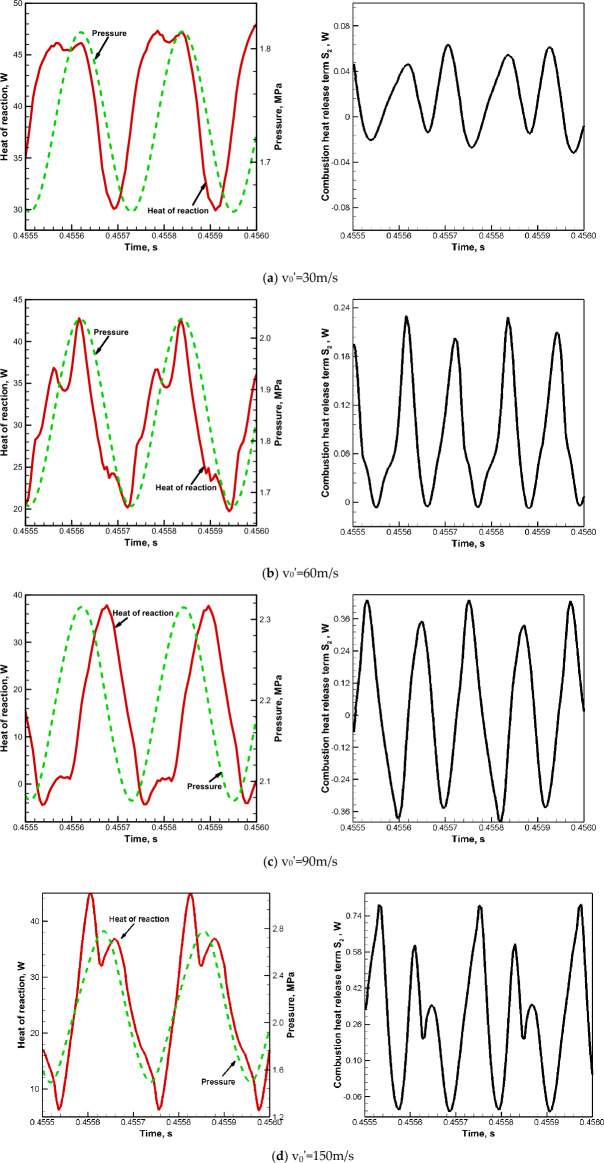
Time histories (left) of area-weighted pressure and heat release rate in the designated integral region and the corresponding combustion heat release terms (right) for different excitement amplitudes.

According to statistics results, the area-averaged pressure and heat release rate in the designated integral region are respectively stable at 1.71 MPa (consistent with the Fig. 6) and 32.60 W without extrinsic excitation, and their oscillation waveforms can be basically ignored compared with what is shown in Fig. 10. It can be seen from the left column of Fig. 10 that the phase differences of the area-weighted pressure and heat release rate in the specified region are respectively − 57.27°, − 8.18°, 90° and 32.73° when v_0_′ = 30 m/s, 60 m/s, 90 m/s and 150 m/s, and the corresponding combustion heat release term S_2_ is presented in the right column of Fig. 10. Taking the transverse velocity disturbance with v_0_′ = 60 m/s as the reference condition, the following analysis is carried out by means of the Rayleigh criterion (see Eq. (15)) and acoustic power balance equation (see Eq. (16)). When v_0_′ = 30 m/s, the excitement amplitude is too small to effectively drive high-temperature combustion gas and promote premixed combustion, and then the phase difference between pressure and heat release rate cannot be eliminated in the designed integral region. As the acoustic excitation generation term S_1_ and the combustion heat release generation term S_2_ are both small, the sustained combustor pressure oscillation amplitude is small in the stable oscillation stage, and the time-averaged pressure is basically unchanged compared with that before the acoustic excitation is started. When v_0_′ = 90 m/s, the larger excitement amplitude results in the faster completion of heat release reaction in the front part of combustor, and even the reverse endothermic reaction occurs locally, which can be proven from the significantly enlarged variation range of heat release rate in left column of Fig. 10c. However, the oscillation waveform of heat release rate lags behind the pressure greatly due to the excessive disturbance velocity. The Rayleigh index and time integral of combustion heat release term S_2_ are basically 0. And it can be seen from Fig. 7 that a smaller drop of the transverse pressure oscillation amplitude along the x direction indicates a larger the acoustic velocity term p′u′. Although the acoustic excitation term S_1_ is larger under this acoustic excitement condition, the sustained combustor pressure oscillation amplitude is relatively small. Besides, the kinetic energy generated by acoustic excitation and internal energy generated by combustion heat release can be used to increase the time-averaged combustor pressure when the pressure oscillation dissipation is small. When v_0_′ = 150 m/s, the variation range of heat release rate decreases, as does the phase difference (32.72°) between area-averaged pressure and heat release rate. Similar phenomena are also observed when the integral region is enlarged to half of the combustion chamber in y-direction (such as (0 ≤ x ≤ 0.348 m, − 0.07 m ≤ y ≤ 0)) for other excitement amplitudes, which means that the combustion reaction has been basically completed in the designed integral region. The time-averaged combustor pressure is basically the same as that of v_0_′ = 90 m/s, and the combustor pressure oscillation amplitude and combustion heat release term promote each other at a rather high level under a large acoustic excitation term, which is more destructive to the engine.

**Figure 11:**
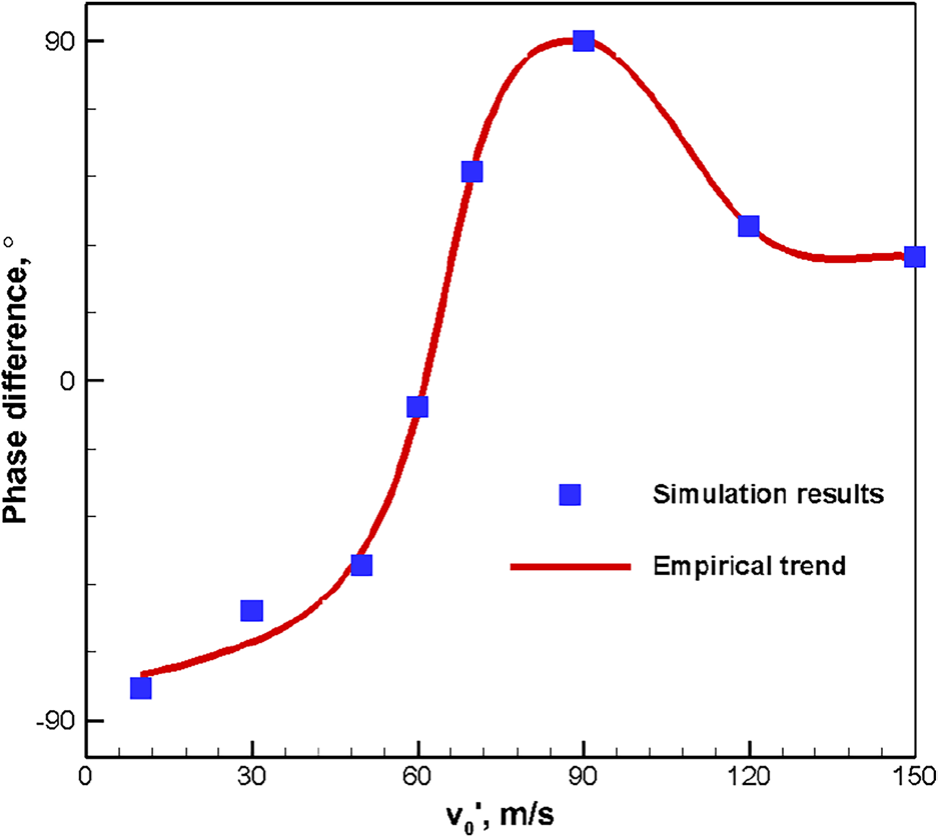
Phase-difference variation trend between area-weighted pressure and heat release rate with respect to excitation amplitude.

Based on the simulation results, the relationship between the excitement amplitude and phase difference of the area-weighted pressure and heat release rate oscillation in the specified region is collected in Fig. 11. With the increase of excitement amplitude, the growth rate of phase difference is slow first and then fast (around v_0_′ = 60 m/s), but there is an upper limit of about 90° (around v_0_′ = 90 m/s). A further increase of excitement amplitude will bring about a downward trend of first rapid and then slow change for the phase difference. Therefore, it is necessary to limit the excitement amplitude to less than 30 m/s.

## Conclusions

In this paper, the Euler–Lagrange method is used to numerically simulate the acoustic response of LOX/GCH_4_ pintle engine under transverse velocity disturbance with different excitement amplitudes, which can provide guidance for the related fire tests. Due to some simplified processing about atomization process, reaction mechanism and artificial acoustic excitement, the simulation results can not truly reflect the spray and combustion flow-field structure within the LOX/GCH_4_ pintle engine. The main conclusions focuses on the trend change of acoustic response: The adopted acoustic excitation method can produce the one-order transverse oscillation response of engine combustor with the excitement frequency. Increasing the excitation amplitude can promote liquid oxygen evaporation and the mixing reaction between oxygen and methane. According to Rayleigh criterion, the acoustic response maintenance mechanism under extrinsic excitation is summarized for pintle engines. Along with the monotone increase of the phase difference between the area-weighted pressure and heat release rate oscillation, the pressure oscillation amplitude and excitement amplitude show a nonlinear relationship. Large-amplitude combustor pressure oscillations is maintained by a large excitement amplitude and the in-phase oscillation of combustion heat release and pressure in combustion reaction region. At last, it should prevent the external excitement from making the phase difference enter the rapid-rise stage.

## Supplementary Information

Supplementary Information.

## Data Availability

The datasets used or analysed during the current study available from the corresponding author on reasonable request.
