# Supplementary material for: Transient spray combustion characteristics in a gas–liquid pintle rocket engine under acoustic excitation
Source: Sci Rep. 2024 Jun 7;14:13135. doi: 10.1038/s41598-024-64027-2 (PMC11637071; doi:10.1038/s41598-024-64027-2)
Supplement: Supplementary file 1 — Supplementary Information. [file 41598_2024_64027_MOESM1_ESM.docx]

The dynamic information **U**(**R**, t) in linear space **R** can be decomposed into time and spatial information using the following equation:

$U\left(R,t\right)=\sum_{m=1}^{M} f_{m}\left(t\right)\cdot\Phi_{m}\left(R\right)$ (A.1)

where **Φ** = [Φ_1_···Φ_m_···Φ_M_] is a set of maximum independent vectors in linear space **R**, i.e. a set of basis in **R** space, Φ_m_ is a mode of **U**(**R**, t) which includes spatial information, F = [*f*_1_···*f*_m_···*f*_M_] is the projection of **U**(**R**, t) on the vector **Φ**, i.e. a linear expression relative to the set of basis in **R** space, *f*_m_(t) is the time coefficient of Φ_m_ and represents the projection of **U**(**R**, t) on the vector Φ_m_ at time t.

It can be assumed that a set of time-discrete data **U** obtained in the unsteady simulations, contains the data, U_1_(**R**), U_2_(**R**)···U_K_(**R**), which corresponds to discrete-time points, t_1_, t_2_···t_K_. The time-discrete data U can be decomposed as

$U^{*}=\Phi\cdot F^{T}=\left[\Phi_{1} \Phi_{2}\cdot\cdot\cdot\Phi_{K}\right]\cdot\left[f_{1} f_{2}\cdot\cdot\cdot f_{K}\right]^{T}$ (A.2)

The error E between the original data **U** and the reconstructed information **U**^*^ is

$E=\left\|U-U^{*}\right\|$ (A.3)

Based on the idea of orthogonal decomposition, no two vectors in a set of basis in **R** space are linearly independent. In order to obtain the set of basis that minimizes the error E, the solution is as follows:

1. Solve the eigenvalue problem of the self-covariance matrix **C** = **U**^T^**U**:

$C\cdot A_{k}=A_{k}\cdot\lambda_{k}$ (A.4)

1. Export the mode Φ_k_ corresponding to the eigenvalue λ_k_ through Equation (A.5):

$\Phi_{k}=\frac{U\cdot A_{k}}{\left\|U\cdot A_{k}\right\|}$ (A.5)

1. Calculate the time coefficients of all modes using the following equation:

$F=\left[\Phi_{1} \Phi_{2}\cdot\cdot\cdot\Phi_{K}\right]^{T}\cdot U$ (A.6)

(4) The magnitude of the eigenvalue λ_k_ reflects the energy contribution of the corresponding mode Φ_k_. All the modes[Φ_1_···Φ_m_···Φ_K_] and their time coefficient [*f*_1_···*f*_m_···*f*_K_] are rearranged in the order of eigenvalues from large to small λ_1_>λ_2_>···>λ_k_.
